# Phytochemical Analysis and Toxicity Study of* Aristolochia paucinervis* Rhizomes Decoction Used in Moroccan Alternative Medicine: Histopathological and Biochemical Profiles

**DOI:** 10.1155/2019/1398404

**Published:** 2019-07-03

**Authors:** Mohammed Bourhia, Ayoub Lahmadi, Hafid Achtak, Ayoub Touis, Jamal Elbrahmi, Riaz Ullah, Abdelaaty A. Shahat, Hafiz Majid Mahmood, Souad Aboudkhil, Laila Benbacer, Naima Khlil

**Affiliations:** ^1^Laboratory of Chemistry Biochemistry, Nutrition, and Environment, Faculty of Medicine and Pharmacy, University Hassan II, Casablanca, Morocco; ^2^Laboratory of Biochemistry, Environment, and Agri-food, Faculty of Science and Technology University Hassan II, Mohammedia, Morocco; ^3^Environment and Health Team, Department of Biology, Multidisciplinary Faculty of Safi, Cadi Ayyad University, Safi, Morocco; ^4^Laboratory of Botany, Faculty of Sciences, University of Abdelmalek Essaadi Tetouan, Morocco; ^5^Medicinal, Aromatic and Poisonous Plants Research Center (MAPRC), College of Pharmacy, King Saud University, PO Box 2457, Riyadh 11451, Saudi Arabia; ^6^Phytochemistry Department, National Research Centre, P.O. Box 1262233, El Bohouth St., Dokki, Giza, Egypt; ^7^Department of Pharmacology, College of Pharmacy, King Saud University PO Box 2457, Riyadh 11451, Saudi Arabia; ^8^Life Science Division, National Centre for Energy, Sciences, and Nuclear Techniques, Rabat, Morocco

## Abstract

**Ethnopharmacological Relevance:**

* Aristolochia paucinervis* (*A. paucinervis*) (Aristolochiaceae) is a plant frequently used in Moroccan alternative medicine. The aim of the current study is to investigate the phytochemical composition of rhizomes decoction of* A. paucinervis* (RDA) and to evaluate its acute and subacute toxicity following the OECD guidelines.

**Materials and Methods:**

The qualitative phytochemical analysis of* A. paucinervis* was performed using standard qualitative phytochemical procedures. The acute toxicity of rhizomes decoction of the studied plant was evaluated in mice at single doses of 1, 2, and 4 g/kg of body weight for 14 days. In subacute toxicity study, the decoction was orally administered to mice at three different doses (0.5, 1, and 1.5 g/kg/day) for 28 days. Histopathological and biochemical parameters were investigated.

**Results:**

The preliminary phytochemical screening showed the presence of flavonoids, saponins, alkaloids, and polyphenols and the absence of anthraquinones, sterols, and terpenes. There was no mortality and no significant changes occurred in animals treated with 1 and 2 g/kg in the acute toxicity model. The signs of toxicity and morbidity were remarkable with the highest tested dose (4g/kg). LD_50_ (dose required to kill 50% of the test population) was determined as 4 g/kg. Repeated oral administration of 1 and 1.5 g/kg/day of RDA for 28 days induced significant disturbance of serum parameters (AST, ALT, LDH, urea, creatinine). Kidney and liver extracted from mice fed with 1 and 1.5 g/kg/day showed significant histopathological injuries as tubular necrosis, inflammatory infiltrate, tubular degeneration, necrosis, and hepatic cholestasis. Meanwhile, neither histopathological nor biochemical alterations were observed in mice treated with 0.5 g/kg/day of body weight in comparison to the control group.

**Conclusion:**

RDA showed toxicity in mice at a dose of 1 g/kg/day under subacute toxicity conditions. RDA is safe at a single dose inferior to 4 g/kg of body weight. The plant extract prepared by decoction showed more poisonous effect than the extract prepared by maceration at room temperature.

## 1. Introduction

Herbs are used throughout the world as an old form of health care. They have played a crucial role in the elaboration of modern medicine such as the conception of synthesized drugs. The medicinal plants have continued to maintain human health for many years [1]. The Moroccans practice alternative medicine for ages and 75% of the Moroccan citizens are relying on traditional medicine as a cure for many ailments [2]. Many people use plants as an alternative medicine to treat diseases because they are considered safer than drugs and ensure an affordable treatment without adverse effects [3]. Not all herbs growing on earth are safe to be used, as a matter of fact, the chemical compounds contained in the plant may be safe for the plant itself but not for humans or animals [4].

*Aristolochia paucinervis* Pomel is a wild species commonly used in Moroccan traditional medicine for the treatment of a wide variety of diseases, such as skin infections and abdominal pain [5]. The powder prepared from the rhizomes is used with salted butter to treat skin injuries, infections, stings, and bites [6, 7]

In Morocco,* A. paucinervis* rhizomes are attracting more attention in ethnomedicine, because of its role in cancer treatment [8]. The traditional healers recommend the use of this species with caution for some weeks only [9]. It was reported that the ingestion of genus* Aristolochia* in traditional treatment of cancer without caution is often accompanied by kidney failure [10]. Although genus* Aristolochia *showed cytotoxic effects against cancer cell lines and apoptosis-induced pathways, its preparation is banned in many countries [11, 12]; even though Morocco has developed the scientific validation of the herbs used in alternative medicine, no more species are validated for quality and safety control [13].

This work was undertaken to screen the phytochemicals of* A. paucinervis *rhizomes decoction and to evaluate its acute and subacute toxicity. Three different doses of RDA were administered by oral gavage to mice. The serum parameters and histopathological changes were investigated.

## 2. Material and Methods

### 2.1. Plant Material

Plant material was collected in December 2016 at 30 km East of Khouribga City, Morocco. The plant was authenticated by Dr. Mohammed Fanane (Department of Botany, Scientific Institute of Rabat, Morocco). Voucher specimens have been preserved in the herbarium of Scientific Institute of Rabat, Morocco, under # 101545. The rhizomes were washed with water and dried at room temperature in a shady and dry ventilated place.

### 2.2. Preparation of Rhizomes Extract

Twenty five grams of dried rhizomes powder was boiled for 20 minutes at 100°C and cooled to room temperature. The solution was filtered and concentrated in a rotary vacuum evaporator to yield 5 g dry extract. The extracted material was suspended in distilled water.

### 2.3. Qualitative Phytochemical Screening

*A. paucinervis *rhizomes extract underwent phytochemical analysis for determining the major classes of secondary metabolites in the plant rhizomes such as polyphenols, alkaloids, saponins, terpenoids, flavonoids anthraquinones, sterols, and terpenes using standard methods as described in the literature [14].

### 2.4. Animal Material

Male adult Swiss mice with an average weight of 25 g were used for the current research. They were purchased from the animal colony of Pasteur Institute, Morocco. Mice were acclimatized in the animal holding with standard conditions; light/dark cycles (12/12 h), temperature (24±2°C) and air changes. Standard pellet diet was freely available to mice.

### 2.5. Toxicological Evaluation of Rhizomes Decoction of* A. paucinervis*

#### 2.5.1. Acute Toxicity Study

The mice were segregated into 4 groups of six mice, including a control group, and then deprived for 12 h of food. The RDA was administrated orally in three doses: 1, 2, and 4 g/kg following OECD, 2008, Guideline No. 425 [15], simultaneously the control group given the same volume of distilled water. The mice were kept under observation during the first day (intermittently for 8 hours); then, every day for 14 days [16], general behavior and clinical symptoms of toxicity were observed.

#### 2.5.2. Subacute Toxicity Study

The mice were segregated into 4 groups of six mice, three treated and one control group. In the treated groups, the RDA was administered repeatedly for 28 days in doses 0.5 g/kg/day, 1 g/kg/day and 1.5 g/kg/day following OECD, 1998, Guideline No. 407 [17]; at the same time the control group was given distilled water. The animals were observed daily and toxic manifestations were registered [18].

#### 2.5.3. Biochemical Parameters

On the head of the experiment period, the experimental animals were subjected to cerebral dislocation for blood collection using laboratory sample tubes. AST, ALT, LDH, urea, and creatinine were the biochemical parameters selected to be determined using an automated analyzer.

#### 2.5.4. Histopathological Evaluation

On the head of the experiment period, the animals were subjected to cerebral dislocation. Liver and kidney were collected for histopathological studies. The tissues were washed and fixed in 10% formaldehyde solution, dehydrated with alcohol and then enclosed in paraplast. Micrometer sections were conducted (5 *μ*m thickness) and maintained with Hematoxylin-Eosin (H&E) for microscopic observation. Tissue sections of organs were examined with a light microscope [19].

### 2.6. Statistical Analysis

Quantitative data were analyzed taking into account the mean ± standard error of the mean (SEM). The significance between means was assessed using one-way ANOVA. Tukey post hoc test was employed for multiple comparisons. Statistically, data showed to be significant when* p* value<0.05.

## 3. Results

### 3.1. Qualitative Phytochemical Analysis

The results of the phytochemical analysis of* A. paucinervis* rhizomes are represented in Table 1.

### 3.2. Acute Toxicity of Rhizomes Decoction of* A. paucinervis*

In the first days of treatment, with a dose of 1 g/kg, a slight behavioral change was recorded in the treated mice, resulting in an accelerated running of 2 to 4 min compared to control group. Clinical symptoms such as diarrhea, lack of appetite, lethargy, salivation, the difficulty of locomotion, reduced activity, and convulsions were observed after animals dosing of doses of 2 and 4 g/kg of body weight. On the other hand, LD_50_ was determined at the highest dose administered of 4 g/kg (three over six mice were dead).

### 3.3. Subacute Toxicity of Rhizomes Decoction of* A. paucinervis*

The clinical symptoms observed in treated animals were carefully registered during the whole period of feeding the RDA. With a dose of 0.5 g/kg, there were no visible toxic effects. We registered difficulty of locomotion, ataxia, restriction of food intake, and reduced activity in both groups given 1 and 1.5 g/kg

### 3.4. Effect of Rhizomes Decoction of* A. paucinervis *on the Mice Weight

**Figure 1:**
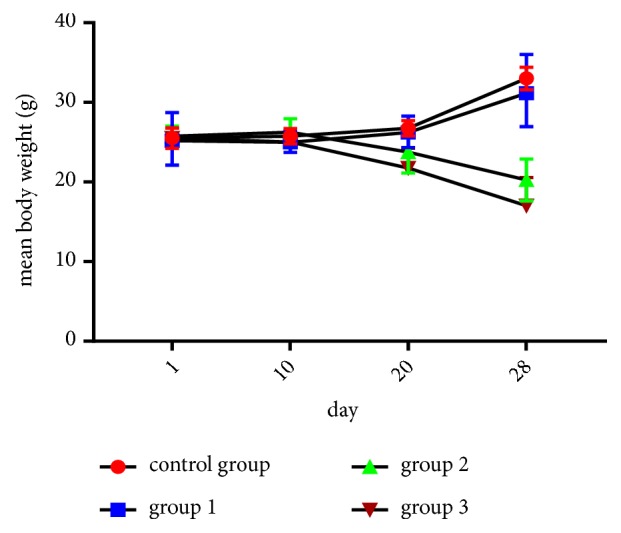
Effect of RDA at doses of 0.5 g/kg/day (group 1), 1 g/kg/day (group 2), and 1.5 g /kg/day (group 3) on the average weight of treated mice (results represent the means ± SEM).

Throughout the whole period of dosing of RDA, there was no significant variation in the weight of fed animals with 0.5 g/kg/day (group 1) in comparison to control group (p>0.05). The fed animals with a dose of 1 g/kg/day (group 2), 1.5 g/kg/day (group 3) induced an important weight loss which began in the first week of dosing and attended to be very significant at the end of the treatment period (Figure 1).

### 3.5. Effect of Rhizomes Decoction of* A. paucinervis *on Biochemical Parameters

**Figure 2:**
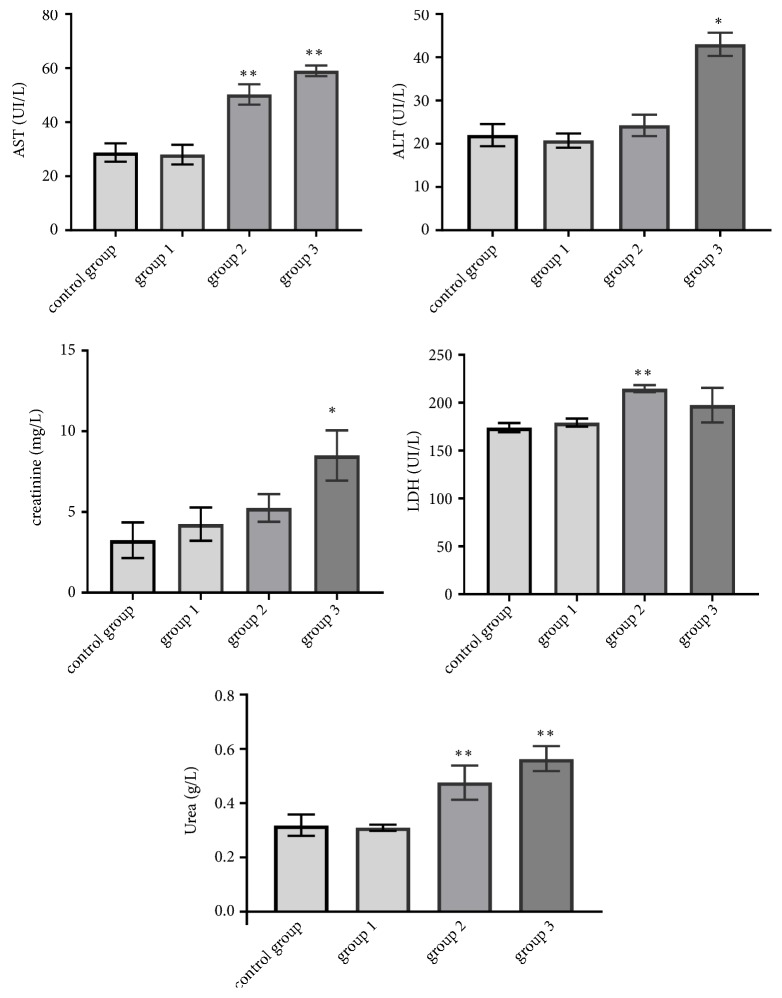
Effect of RDA on AST, ALT, LDH, urea, and creatinine after 28 days of treatment (group 1: 0.5 g/kg/day; group 2: 1g/kg/day; group 3: 1.5 g/kg/day) (results represent the means ± SEM).

The results of biochemical parameters were examined, including ALT, AST, urea, creatinine, and LDH, represented in Figure 2.

The findings of biochemical parameters showed a significant augmentation of AST measured in treated group 2 (1 g/kg/day) and 3 (1.5 g/kg/day) in comparison to control group (p<0.05). There was no significant change remarked in group 1 fed with the lowest dose (0.5 g/kg/day) (p>0.05) (Figure 2; Table 2). Regarding ALT transaminases, there was no significant elevation in groups 1 and 2 fed with 0.5 g/kg/day and 1 g/kg/day, respectively, in comparison to the control group; meanwhile, we noted a significant increase in group 3 (1.5 g/kg/day) (p<0.05) (Figure 2; Table 2). Considering the markers of kidney function, the concentration of creatinine significantly rose in group 3 (1.5 g/kg/day) in comparison to the control group (p<0.05). No significant elevation noted in group 1 (0.5 g/kg/day) and 2 (1 g/kg/day) (Figure 2; Table 2). Concerning urea, the findings showed insignificant variations in groups 1 (0.5 g/kg/day) and 2 (1 g/kg/day) in comparison to the control group (p>0.05). There was a significant augmentation occurred in group 3 fed with 1.5 g/kg/day (p<0.05) (Figure 2; Table 2). Concerning LDH, no significant changes were detected in groups 1 and 3 fed with 0.5 g/kg/day and 1.5 g/kg/day respectively in comparison to the control group (p>0.05). Meanwhile, a significant accentuation was remarked in group 2 fed with 1 g/kg/day (p<0.05) (Figure 2; Table 2)

### 3.6. Histopathological Alterations

#### 3.6.1. Kidney

The histopathological injuries detected in kidney tissues of treated mice, such as tubular necrosis, hyaline necrosis, inflammatory cell infiltration, and cell congestion, were the main tissue damage which became very detectable in the kidney of the mice fed with increasing doses of RDA as shown in Table 3.

Considering the major histopathological alterations detected in the kidney of treated mice, such as tubular necrosis, hyaline necrosis, inflammatory cell infiltration, and vascular dilatation, no significant difference was shown between control and fed mice with 0.5 g/kg/day (group 1), nor 1 g/kg/day (group 2). For comparisons between treated groups in terms of announced histopathological injuries, no significant difference was remarked between group 1 (0.5 g/kg/day) and group 2 (1 g/kg/day) (p>0.05). Considering groups 2 and 3, a significant difference for vascular dilatation injury was observed (Tables 3 and 4) (p<0.05).

**Figure 3:**
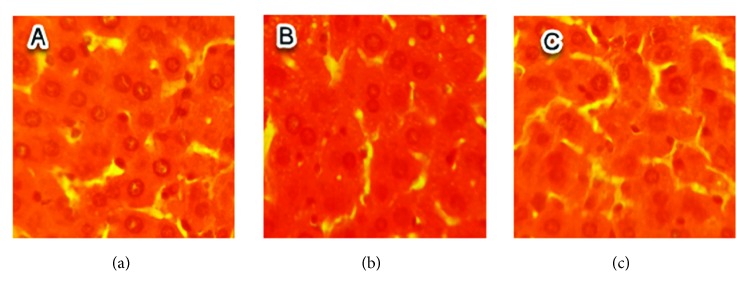
Sections of kidney parenchyma of control mice (H&E x 40). (a), (b), and (c) are kidney sections of normal tissues of three mice selected randomly from the control group.

**Figure 4:**
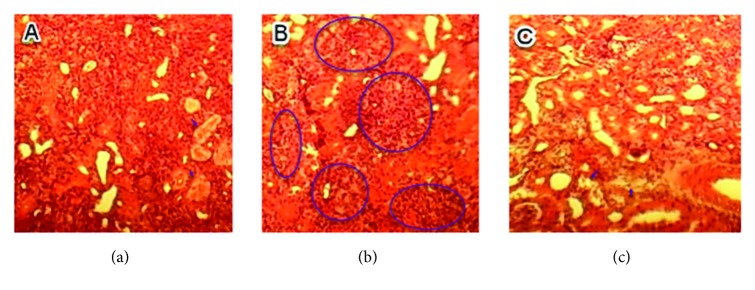
Sections of kidney parenchyma of mice fed with 1 g/kg/day (H&E, x 40). (a) Tubular necrosis; (b) inflammatory infiltrate; (c) tubular degeneration.

**Figure 5:**
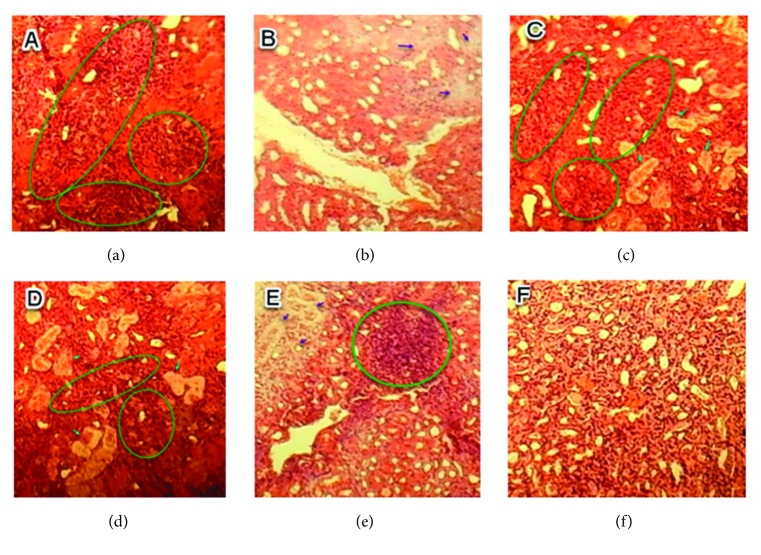
Sections of kidney parenchyma of mice fed with 1.5 g/kg/day (H&E, x40). (a) Inflammatory infiltrate (circle); (b) renal necrosis; (c) inflammatory infiltrate (circle) and tubular necrosis (arrow); (c) tubular degeneration (arrow) and inflammatory infiltrate (circle); (e) inflammatory infiltrate (circle) and tubular necrosis (arrow); (f) cell congestion.

No histopathological injuries were detected in the kidney of fed animals with 0.5 g/kg in comparison to the control group (Figure 3). However, for groups treated with doses of 1 and 1.5 g/kg, nephropathy, tubular necrosis, coagulation, necrosis, inflammatory infiltrate, alteration of architecture, tubular degeneration, and cellular congestion were the main histopathological changes detected which are summarized in Figures 4 and 5.

#### 3.6.2. Liver

The histopathological changes recorded in liver tissue of treated animals, such as hepatocellular necrosis, lobular necrosis, cell vacuolization, and inflammatory infiltrate, were the mean tissue damage, which became very remarkable in the liver of fed mice with increasing doses of RDA as reported in Table 5.

**Figure 6:**
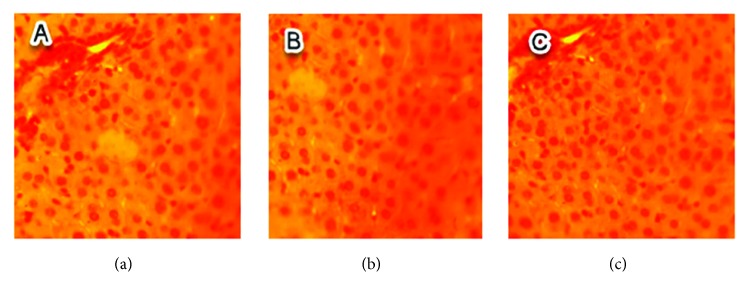
Sections of liver parenchyma of control mice (H&E, x40). (a), (b), and (c) are liver sections of normal tissues of three mice selected randomly from the group of control mice.

**Figure 7:**
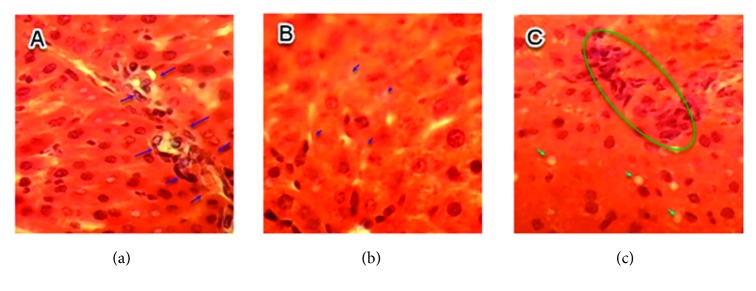
Sections of liver parenchyma of fed mice with 1 g/kg/day (H&E, x40). (a) Inflammatory infiltrates; (b) hepatic necrosis; (c) hepatic necrosis (arrow) and inflammatory infiltrates (circle).

**Figure 8:**
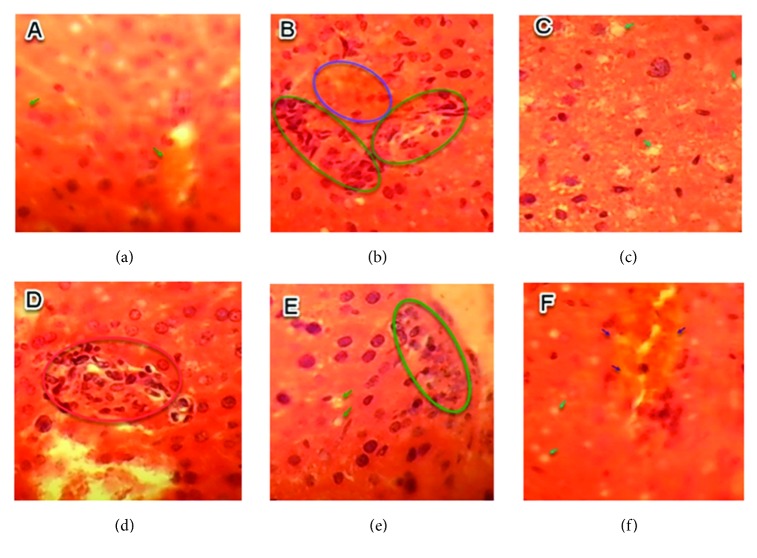
Histologic section of liver tissue of treated mice with 1.5 g/kg/day (H&E, x40). (a) Hepatic cholestasis; (b) inflammatory infiltrate (green circle) and hepatic cholestasis (blue circle); (c) hepatic necrosis; (d) inflammatory infiltrate; (e) hepatic necrosis (arrow) and inflammatory infiltrate (circle); (f) hepatic cholestasis (blue arrow) and hepatic necrosis (green arrow).

For the lowest dose tested (0.5 g/kg/day), no histopathological changes were detected in the liver compared to the control group (Figure 6), while for the groups treated with 1 and 1.5 g/kg/day, the most histopathological changes are summarized in Figures 7 and 8.

## 4. Discussion

Since ancient time, people believe that the use of herbal medicines is safe because of their natural origin. In Morocco, there are no regulatory requirements for the majority of medicinal plants sold by herbalist, including adherence to information in pharmacopeias [20]. The present study focused on the medicinal plant of* A. paucinervis *which has extensively been used in Moroccan alternative medicine for its medicinal properties.

Studying the acute toxicity of RDA was useful to assess its toxicity at single doses. As reported in the literature, there are very little reported cases of acute human or animal poisoning by* Aristolochia *species [21]; however, plants of Aristolochiaceae family are considered as most dangerous herbs when consumed for a long period due to their content of aristolochic acids (AAs). The distribution of these compounds appears to be homogenous within the genus and could here account for the strongly positive reaction observed for alkaloids with Dragendorff's reagent during our phytochemical screening [22, 23]. The findings of acute toxicity revealed that the mortality was remarked in treated animals with the great dose only (4g/kg) responsible for 50% of animals mortality. Therefore, the rhizomes decoction of* A. paucinervis* could be classified as poorly toxic in single administration [24]. Concerning the acute toxicity of RDA, no correlation has been established between LD_50_ and AAs contents [25]; hence, the aristolochic acids may be not responsible for acute toxicity reported in the present work.

The clinical symptoms occurred in mice under subacute toxicity conditions like convulsions, the difficulty of locomotion, weight loss, and hypoactivity are probably related to toxic properties of RDA [26]. The biochemical markers of kidney evaluate the normal renal function. They attest the glomerular filtration rate. Renal dysfunction is often indicated by an increase or decrease of markers concentrations [27].

Urea is the final nitrogenous excretion derived from protein amino acid catabolism and it is produced in the liver. In kidney the urea is filtered from serum, it is the common biochemical indice measured for evaluating kidney function on the basis of urea concentration in serum, and it is largely used for the diagnosis of acute kidney failure [28]. In this current research work, the abnormal increase of urea in the serum of treated mice is probably related to the toxic effect of RDA on the kidney. Creatinine is a normal metabolic waste produced by the body. It is a breakdown product derived from creatinine phosphate in muscle, eliminated mainly by the kidney. The creatinine rate in the body depends on the renal elimination capacity and muscle mass [29]. The renal failure diagnosis is commonly indicated when the concentration of plasma creatinine is higher than the value of reference [30]; hence, the significant increase of creatinine in the serum of treated mice in our study bears witness of the probable toxic effect of RDA on kidneys.

Transaminases (ALT, AST) are enzymes found inside living cells, especially in the liver and muscles which intervene in a multitude of biological reactions [31]. The dosage of ALT and AST is used to indicate a problem in the liver; their increase in the serum is due to an abnormal release induced by damaged liver cells under intoxication or diseases occurring in the liver [32]. Considering the results of ALT and AST reported in this study, the significant increase in their activities in the serum of treated mice is frequently related to hepatic cytolysis effect of RDA.

Lactate dehydrogenase (LDH) is a biomarker of tissue damage because this enzyme is normally found in most tissues of the organism and only in small amounts in the blood circulatory. When the tissues are damaged, the cells release the LDH causing an increase in its concentration in the blood circulatory. The dosage of LDH is generally required in the diagnosis of tissue lesions [33]; the significant increase of serum activity of LHD registered in our work indicates a probable toxic effect induced by RDA on global tissues of treated mice.

The histopathological injuries reported in the kidney of treated animals in this current work such as nephropathy, necrosis, tubule-interstitial fibrosis, inflammatory infiltrate, altered tissue architecture, tubular degeneration, tubular necrosis, and cell congestion are in confirmation with the biochemical alterations of urea and creatinine, and even the histologic changes observed in liver tissue, such as necrosis, lymphocytic infiltrate, hepatic cholestasis, tubular necrosis, foci of hemorrhage, cell congestion, and altered tissue architecture, are in accordance with serum parameters variation registered in this research work (AST, ALT, LDH).

The histopathological and biochemical findings reported in the present study were in confirmation with kidney failure occurring in Belgium patients adopting weight loss regimes including species of* A. fangchi*. It was reported that interstitial renal fibrosis induced has been attributed to aristolochic acids contained in some plants used in Chinese folk medicine [34].

The obtained results in the present work were comparable to those reported in the literature [35, 36]. It was reported that the aqueous extract of* A. longa* prepared by maceration at room temperature has a negative effect on the biochemical parameters and histopathological tissues under acute toxicity conditions with oral administration of a dose no lower than 2.5 g/kg. Meanwhile, our results showed that the alterations of biochemical parameters and histopathological tissues occurred in mice only with oral gavage of 1 g/kg/day of body weight of the same plant extract prepared by decoction. Therefore, the decoction used in the preparation of natural remedies could increase the risk of plants toxicity compared to maceration at room temperature. Moreover, reports showed that the prepared extract from* A. manushuresis *induced hepatic necrosis in mice fed with 2 g/kg/day [24]. Aristolochiae Fructus aqueous extract was responsible for involving nephrotoxicity in mice according to dose-dependent manner [18].

Aristolochic acids contained from genus* Aristolochia* are responsible for renal damage [37].* A. paucinervis* belongs to this chemically homogenous genus and is therefore likely to contain Aristolochic acids. These chemical compounds are responsible for the nephrotoxic effects [38, 39]. AAs spring up from* Aristolochia* spp found in two carboxylic acids such as aristolochic acid I (AAI) and aristolochic acid II (AAII) [40]. For performing comparison regarding aristolochic acids toxicity, the AAI was found to be more toxic than AAII, and there are other structural analogues having less or no toxicity [41].

Biologically, aristolochic acids open experimental pathways for conceptualizing the mechanism by which the renal fibrogenesis is involved in human pathology. The identification of complications related to treatment with medicinal plants puts several questions in terms of health safety [42]. In this way, many types of research have established a relationship between reactive metabolites formed during the bioactivation of herbal constituents and their toxicity. The toxic effect of RDA on the liver could result in the fact that this organ is the first target of toxicity. The liver-renal toxicity could be induced by both AAI and AAII subjected to reactions leading to a reduction of the nitro group to reactive cyclic nitrenium ions which create covalent DNA adducts with the exocyclic amino groups [43]; this is provoking cell cycle arrest [44, 45]. AAI was found more toxic than AAII; however, other structural analogues were found very less toxic [41]. Therefore, tissue destruction shown in the current research could be induced by high and continuous activation of the immune system undergoing AAs metabolite effects [35].

## 5. Conclusion

This study showed that the rhizomes decoction of* A. paucinervis *was slightly toxic in single dose oral administration of less than 4 g/kg, while it seems to be more toxic at a dose of 1 g/kg under the conditions of subacute toxicity. The decoction used in the preparation of natural remedies increases the risk of the poisonous effect of the plant, in comparison with the maceration at room temperature. Based on this study, it is crucial to pay more attention to herbs used in traditional medicine for safety control and the preparation method and not to consider herbs as always GRAS (Generally Recognised As Safe).

## Figures and Tables

**Table 1:** Phytochemical analysis of *A. paucinervis* rhizomes.

Polyphenol	Alkaloids	Flavonoids	Anthraquinone	Sterols and terpene	Saponins
++	+++	++	-	-	+

+++: strong positive test; ++: positive test; +: low positive test; *–*: negative test.

**Table 2:** Results of Tukey test for comparisons between groups regarding biochemical parameters variation (Subacute Toxicity).

Chemical parameters	control group vs. group 1	control group vs. group 2	control group vs. group 3	group 1 vs. group 2	group 1 vs. group 3	group 2 vs. group 3
AST	0.99	0.001*∗*	0.001*∗*	0.001*∗*	0.001*∗*	0.99
ALT	1	0.98	0.02*∗*	0.004*∗*	0.003*∗*	0.005*∗*
Urea	0.99	1	0.00*∗*	0.99	0.00*∗*	0.00*∗*
Creatinine	0.98	0.96	0.03*∗*	0.99	0.05	0.06
LDH	0,85	0.01*∗*	0.53	0.01*∗*	0.83	0.81

Control: normal diet; group 1: normal diet + 0.5 g/kg/day extract; group 2: normal diet + 1 g/kg/day extract; group 3: normal diet + 1.5 g/kg/day extract.

*∗*: significant at 5% level.

**Table 3:** Lesion scores of the kidney for all groups analyzed using ANOVA test (subacute toxicity).

Histopathology of kidney	Histopathology scores of observation				ANOVA test for global comparison of organ lesions among groups
	(mean) per each group				
	control group	group 1	group 2	group 3	
Tubular necrosis	0	0	0.11±0.01	0.25±0.05	0.006*∗*
Hyaline necrosis	0	0	0.05±0.05	0.19±0.04	0.04*∗*
Coagulation necrosis	0	0	0	0.22±0.17	0.31
Inflammatory infiltrate	0	0	0.35±0.15	0.60±0.10	0.02*∗*
Extended coagulation	0	0	0	0	1
Altered tissue architecture	0	0	0	0	1
Cell vacuolization	0	0	0	0.25±0.25	0.47
Tubular degeneration	0	0	0.20±0.20	0.31±0.19	0.28
Cell congestion	0	0	0.10±0.03	0.17±0.02	0.04*∗*
Tubular atrophy	0	0	0	0	1
Interstitial fibrosis	0	0	0	0	1
Vascular dilation	0	0	0	0.16±0.03	0.006*∗*

Control: normal diet; group 1: normal diet + 0.5 g/kg/day extract; group 2: normal diet + 1 g/kg/day extract; group 3: normal diet + 1.5 g/kg/day extract.

*∗*: significant at 5% level.

**Table 4:** Results of Tukey test for comparisons between groups regarding lesions in the kidney (subacute toxicity).

Histopathology of kidney	control group vs. group 1	control group vs. group 2	control group vs. group 3	group 1 vs. group 2	group 1 vs. group 3	group 2 vs. group 3
Tubular necrosis	1	0.19	0.008*∗*	0.11	0.008*∗*	0.58
Hyaline necrosis	1	0.73	0.049*∗*	0.73	0.049*∗*	0.19
Coagulation necrosis	1	1	0.38	1	0.38	0.38
Inflammatory infiltrate	1	0.15	0.03*∗*	0.15	0.03*∗*	0.33
Extended coagulation	1	1	1	1	1	1
Altered tissue architecture	1	1	1	1	1	1
Cell vacuolization	1	1	0.35	1	0.35	0.53
Tubular degeneration	1	0.74	0.47	0.74	0.47	0.93
Cell congestion	1	0.22	0.06	0.22	0.06	0.52
Tubular atrophy	1	1	1	1	1	1
Interstitial fibrosis	1	1	1	1	1	1
Vascular dilation	1	1	0.009*∗*	1	0.009*∗*	0.009*∗*

Control: normal diet; group 1: normal diet + 0.5 g/kg/day extract; group 2: normal diet + 1 g/kg/day extract; group 3: normal diet + 1.5 g/kg/day extract. *∗*: significant at 5% level.

**Table 5:** Lesion scores of the liver for all groups analyzed using ANOVA test (subacute toxicity).

Histopathology of liver	Histopathology scores of observation (mean) per each group				ANOVA test for global comparison of organ lesions among groups
	control group	group 1	group 2	group 3	
Hepatocellular necrosis	0	0.001±0.0007	0.45±0.05	0.35±0.05	0.002*∗*
Lobular necrosis	0	0	0.13±0.01	0	0.00*∗*
Altered tissue architecture	0	0	0	0	1
Cell vacuolization	0	0	0.02±0.02	0.25±0.05	0.008*∗*
Cell congestion	0	0	0	0	1
Inflammatory infiltrate	0	0.01±0.01	0.17±0.12	0.5±0.10	0.03*∗*
Dilatation of sinusoids	0	0	0	0	1
Kupffer cell hypertrophy	0	0	0	0	1
Hepatic steatosis	0	0	0	0	1
Cholestasis	0	0	0.17±0.17	0.35±0.05	0.13
Haemorrhagic foci	0	0	0	0	1
Disruption of blood vessel	0	0	0	0	1

Control: normal diet; group 1: normal diet + 0.5 g/kg/day extract; group 2: normal diet + 1 g/kg/day extract; group 3: normal diet + 1.5 g/kg/day extract.

*∗*: significant at 5% level.

## Data Availability

All data are available in the following labs: Laboratory of Chemistry Biochemistry, Nutrition, and Environment, Faculty of Medicine and Pharmacy, University Hassan II, Casablanca, Morocco, Laboratory of Biochemistry, Environment, and Agri-food, Faculty of Science and Technology University Hassan II, Mohammedia, Morocco, Environment and Health Team, Department of Biology, Multidisciplinary Faculty of Safi, Cadi Ayyad University, Safi, Morocco, Laboratory of Botany, Faculty of Sciences, University of Abdelmalek Essaadi Tetouan, Morocco, and Life Science Division, National Centre for Energy, Sciences, and Nuclear Techniques, Rabat, Morocco.
